# Association of cerebral white matter hyperintensities with coronary artery calcium in a healthy population: a cross-sectional study

**DOI:** 10.1038/s41598-022-25654-9

**Published:** 2022-12-13

**Authors:** Jinyoung Choi, Jung Youn Kim, Heon-Ju Kwon, Hye Jeong Choi, Sang Heum Kim, Sinae Kim, Jungbin Lee, Ji Eun Park

**Affiliations:** 1 Department of Radiology, Kangbuk Samsung Hospital, Sungkyunkwan University School of Medicine, Seoul, 03181 Republic of Korea; 2 Department of Radiology, CHA Bundang Medical Center, CHA University, Seongnam, Gyeonggi-Do 13496 Republic of Korea; 3 Division of Biostatistics, Department of R&D Management, Kangbuk Samsung Hospital, Sungkyunkwan University School of Medicine, Seoul, 03181 Republic of Korea; 4 Department of Radiology, Soonchunhyang University Bucheon Hospital, Bucheon, Gyeonggi-Do 14584 Republic of Korea; 5 Department of Radiology and Research Institute of Radiology, University of Ulsan College of Medicine, Asan Medical Center, Seoul, 05505 Republic of Korea

**Keywords:** Biomarkers, Diseases, Health care, Medical research, Neurology, Risk factors

## Abstract

In brain magnetic resonance imaging (MRI), white matter hyperintensity (WMH) is a commonly encountered finding and is known to reflect cerebral small vessel disease. The aim of our study was to investigate the association of coronary artery calcium (CAC) with WMH and elucidate the relationship between WMH and atherosclerotic risk factors in a large-scale healthy population. This retrospective study included 1337 individuals who underwent brain MRI and CAC scoring computed tomography at healthcare centers affiliated with a tertiary hospital. Cerebral WMH was defined as Fazekas score greater than 2 on brain MRI. Intracranial artery stenosis (ICAS) was also assessed and determined to be present when stenosis was more than 50% on angiography. The associations of risk factors, CAC score, and ICAS with cerebral WMH were assessed by multivariable regression analysis. In multivariable analysis, categories of higher CAC scores showed increased associations with both periventricular and deep WMHs in a dose-dependent relationship. The presence of ICAS was also significantly related to cerebral WMH, and among the clinical variables, age and hypertension were independent risk factors. In conclusion, CAC showed a significant association with cerebral WMH in a healthy population, which might provide evidence for referring to the CAC score to identify individuals with risk of cerebral WMH.

## Introduction

White matter hyperintensity (WMH) is a commonly encountered finding on T2-weighted and fluid-attenuated inversion recovery (FLAIR) sequences of brain magnetic resonance imaging (MRI)^1,2^. Although the exact pathophysiologic mechanisms of WMH are unclear, it has been shown to be associated with atherosclerotic risk factors such as aging, hypertension, diabetes, smoking and obesity, supporting the contribution of vascular mechanisms to the development of WMH^3–10^. Pathological studies also revealed that WMH is caused by impaired vascular integrity, thus supporting that WMH is a reflection of small vessel disease in the brain^11^. In addition, WMH is clinically important as it has been shown to affect the incidence and prognosis of various neurologic disorders including cognitive decline, dementia, depression, gait disturbance, and stroke^12–23^.

The coronary artery calcium (CAC) score is regarded as a convenient and reliable indicator of atherosclerosis that measures an individual’s cumulative exposure, and it has been shown to be associated with ischemic stroke and cranial artery stenosis as well as coronary heart disease^24,25^. Cerebral small vessel disease is prone to coexist with atherosclerosis of large intracranial arteries, as small perforator vessels supplying white matter arise from the large basal arteries^26–28^. Many studies have revealed a relationship between WMH and atherosclerotic risk factors or carotid artery atherosclerosis; however, only a few studies have focused on the relationship between CAC burden and WMH, and these studies were performed exclusively in elderly or male individuals^29–32^.

With the improved accessibility to neuroimaging in recent years, the high prevalence and clinical importance of WMHs have been increasingly recognized as a predictor of cognitive decline and stroke outcomes^19–23^. This study was motivated by the idea that if the CAC score can be used in clinical practice for predicting the risk of WMH, which is a prognostic factor for various neurologic disorders, it can be a convenient and useful tool for identifying individuals who might benefit from additional studies such as brain MRI^19–23^. We hypothesized that WMH would show strong association with CAC burden, an indicator of atherosclerosis, in a large cohort of healthy individuals from the general population. In addition, we sought to contribute to understanding the mechanism of WMH development by identifying relevant clinical risk factors. Thus, the primary aim of this study was to investigate the association of CAC with WMH in a healthy population. Second, the purpose of this study was to elucidate the relationship between WMH and risk factors for atherosclerosis.

## Methods

### Study population

This study was a general population-based, cross-sectional, retrospective study. We searched the electronic database of participants who underwent health check-ups, including brain MRI and magnetic resonance angiography (MRA), at the Total Healthcare Center of Kangbuk Samsung Hospital in Seoul and Suwon between January 2016 and December 2019 and identified 3983 consecutive adult participants. The study population consisted of examinees who underwent computed tomography (CT) for CAC scoring and brain imaging as a part of comprehensive health check-ups, which are common health screening methods in Korea. For reference, all employees in Korea are required by law to undergo regular annual or biennial health examinations, so many of the participants were employees of various companies or local governmental organizations or family members of employees.

**Figure 1:**
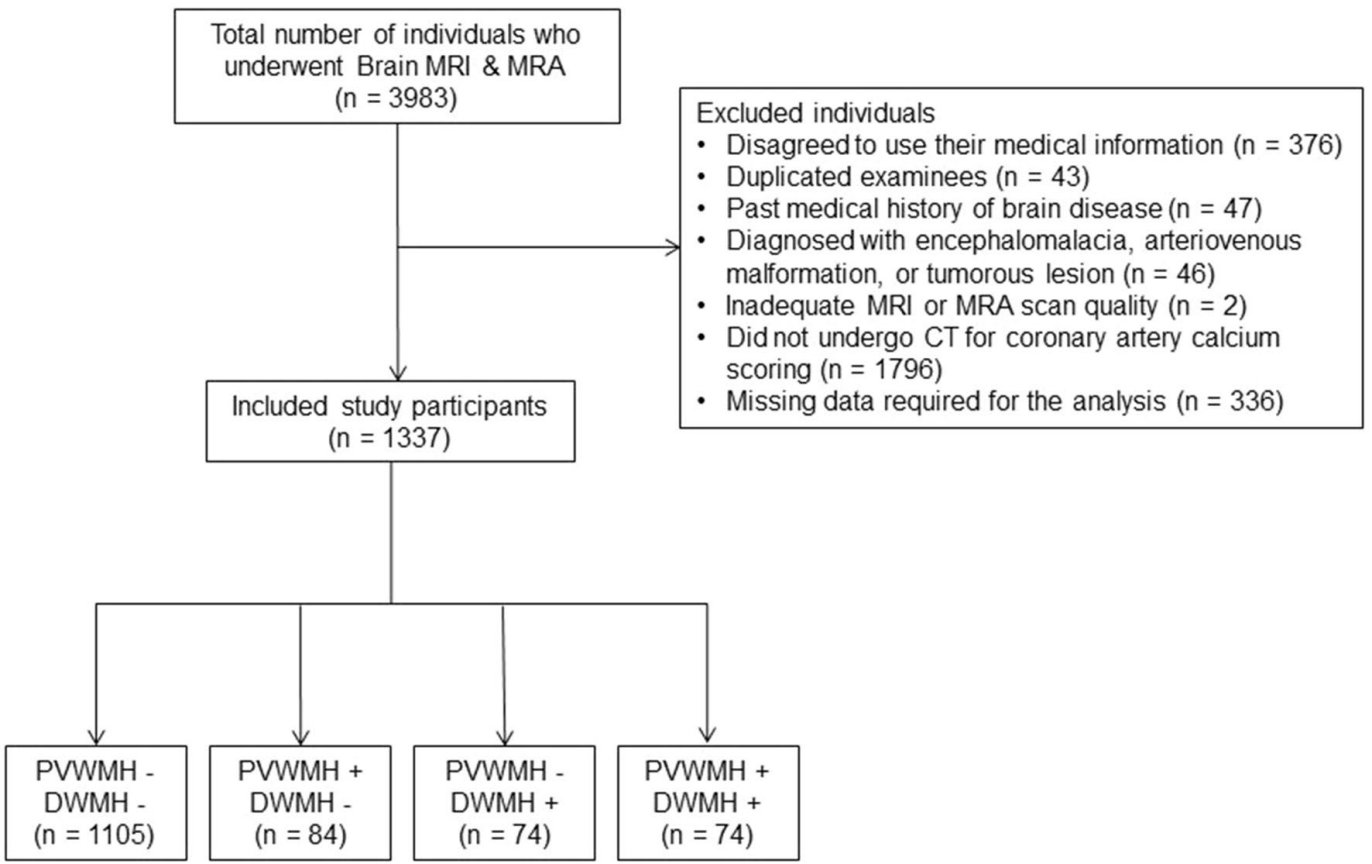
Flow diagram of the included participants. *MRI* Magnetic resonance imaging, *MRA* Magnetic resonance angiography, *PVWMH* Periventricular white matter hyperintensity, *DWMH* Deep white matter hyperintensity.

Of the 3983 individuals, 2646 were excluded for the following reasons: *(a)* individuals who did not consent to the use of medical information for any research purpose in the self-administered questionnaires conducted before the examination (n = 376); *(b)* individuals with duplicated tests were excluded if they underwent repeated examinations during the study period (n  =  43), and CAC scoring CT and brain imaging performed on the same day or at the nearest time interval were selected for the study; *(c)* individuals with a known medical history of dementia, Parkinson’s disease, hydrocephalus, previous brain surgery, brain tumor, Moyamoya disease, stroke or hemorrhage (n = 47); *(d)* individuals detected with significant cerebral pathology during the image analysis, such as encephalomalacia resulting from a previous stroke (measuring larger than 15 mm in diameter) or an old traumatic hemorrhage, an arteriovenous malformation, or a tumorous lesion (n = 46); *(e)* individuals with MRI or MRA scans with inadequate quality for image analysis (n = 2); *(f)* individuals who did not undergo CAC scoring CT (n = 1796); and *(g)* individuals with missing numeric data required for the analysis, including body mass index (BMI) and homocysteine level (n = 336). A flow diagram of the included study participants is shown in Figure 1.

Therefore, 1337 consecutive individuals (mean age, 51.63 ± 9.20 years old; age range, 20–89 years; 1157 [86.54%] male patients) were included in the study. The clinical and imaging findings of all participants were retrospectively assessed. This study was conducted in accordance with the principles of the Declaration of Helsinki and was approved by the institutional review board (IRB) of Kangbuk Samsung Hospital (IRB No. 2020-12-036-006). The requirement to obtain informed consent was waived by the IRB of Kangbuk Samsung Hospital due to the use of deidentified data and the retrospective study design. All study methods were performed in accordance with relevant guidelines and regulations.

### Clinical assessment

We collected the clinical data of the individuals, including sex, age, BMI, systolic and diastolic blood pressure, smoking history, physical activity, and diagnosis and treatment of hypertension, diabetes, hyperlipidemia and coronary artery disease. From the standardized, self-administered questionnaires, we collected data on each individual’s medical and smoking histories and whether they regularly engaged in more than 10 min of vigorous exercise at least 3 times per week.

Since all the participants’ examinations were conducted by appointment at the Total Healthcare Center of Kangbuk Samsung Hospital, laboratory tests were conducted after 12 h of fasting on the same day as brain MRI and MRA and data including glucose, glyco hemoglobin (HbA1c), total cholesterol, low-density lipoprotein cholesterol, high-density lipoprotein cholesterol, triglyceride and homocysteine levels were collected.

Hypertension was defined as current use of antihypertensive drugs, systolic blood pressure ≥ 140 mmHg or diastolic blood pressure ≥ 90 mmHg^33^. Diabetes was defined as current use of antidiabetic medication, a fasting glucose level ≥ 126 mg/dL or an HbA1c level ≥ 6.5%^34^. Dyslipidemia was defined as current use of lipid-lowering agents, total cholesterol ≥ 240 mg/dL, low-density lipoprotein cholesterol ≥ 160 mg/dL, high-density lipoprotein cholesterol < 40 mg/dL or triglycerides ≥ 200 mg/dL^35^.

### Brain MRI & MRA assessments

All individuals underwent brain MRI and MRA using a 1.5-T MRI scanner (Optima MR360, GE Healthcare, Milwaukee, WI or Signa HDxt, GE Healthcare, Milwaukee, WI) using an eight-channel head coil. The imaging protocol included axial T1-weighted images (repetition time [TR]/echo time [TE] = 417–450/9 ms or 400–450/10 ms), T2-weighted images (TR/TE = 4343–4694/100–110 ms or 4084–4494/95–104 ms), FLAIR images (TR/TE = 11,000/127–138 ms or 8800/128–130 ms), and three-dimensional time-of-flight (TOF) MRA images (TR/TE = 28/7 ms or 27/3 ms, slice thickness = 1.2 mm). The slice thickness of all imaging protocols, except TOF MRA, was 5 mm.

The degree of periventricular and deep WMHs were rated separately according to the Fazekas scale^1^ for each subject, as shown in Supplementary Fig. 1 online. PVWMH was scored as follows: 0 = absence, 1 = caps or pencil-thin lining, 2 = smooth halo, and 3 = irregular periventricular hyperintensities extending into the deep white matter. DWMH was classified as follows: 0 = absence, 1 = punctate foci, 2 = beginning confluence of foci, and 3 = large confluent areas. Since cerebral WMH grade 2 or higher is known to be clinically relevant as it is prone to be symptomatic and progressive, we categorized subjects with Fazekas scores of 2 and 3 into the PVWMH and DWMH groups^36,37^.

Intracranial artery stenosis (ICAS) was defined as more than 50% stenosis of the intracranial arteries by TOF MRA analysis based on the Warfarin-Aspirin Symptomatic Intracranial Disease (WASID) method^38^. The included analyzed vessels were the internal carotid artery from the cavernous segment, up to the M2 segment of the middle cerebral artery, the A2 segment of the anterior cerebral artery, the P2 segment of the posterior cerebral artery, the basilar artery, and intracranial segments of the vertebral arteries.

All radiological assessments were performed by a neuroradiologist (J.Y. K.) who was blinded to all clinical and laboratory data. The interobserver reliability of the visual scales was evaluated with the assessment of 700 randomly selected subjects by a second trained radiologist (J.Y. C.), and the intraobserver reliability was assessed with more than a 2-month interval after the first reading. The visual assessment of PVWMH, DWMH, and ICAS showed good interrater (Cohen’s weighted kappa: 0.7, 0.81, and 0.67, respectively; n = 700) and intrarater (Cohen’s weighted kappa: 0.92, 0.88, and 0.65, respectively; n = 1339) agreement.

### Coronary calcium score assessment

The CAC score was evaluated for individuals who underwent CT for CAC scoring within 5 years from the time that brain MRI and MRA were performed^39^. Of the 1337 individuals, 686 underwent brain imaging on the same day, and 651 underwent brain imaging on another day within 5 years.

CAC was detected with a LightSpeed VCT XTe-64-slice multidetector CT (GE Healthcare, Tokyo, Japan) at both the Seoul and Suwon Centers using the same scanning protocol of 2.5 mm thickness, 400 ms rotation time, 120 kV tube voltage and 124 mAs (310 mA × 0.4 s) tube current under electrocardiogram-gated dose modulation. The CAC score was calculated from the 4 major epicardial coronary arteries (left main, left anterior descending, left circumflex and right coronary arteries) according to Agatston et al.^40^. The technicians who performed CT were blinded to any subject information, and the CAC score was automatically determined using HEARTBEAT-CS software (Philips, Cleveland, Ohio, USA). The CAC scores were categorized into three groups: 0, 1–100, and > 100.

### Statistical analysis

The baseline characteristics between subjects with and without cerebral WMH were compared using the χ^2^ test for categorical variables and Student’s *t* test or the Mann‒Whitney test for continuous variables, as appropriate. Variables with a normal distribution are presented as the mean ± standard deviation, while nonnormally distributed variables are presented as the median and interquartile range. Dummy variables were introduced for missing values for the categorical variables.

Multivariable logistic regression analyses were conducted, and odds ratios (ORs) with 95% confidence intervals (CIs) were calculated to evaluate the relationship between cerebral WMH and CAC scores and atherosclerotic risk factors. Since the prevalence of WMH increases with age and varies with sex, adjustment for age and sex was applied in all performed multivariable analyses to evaluate the association between other variables and WMH^18^. Different multivariable logistic regression models were used to assess whether the CAC score has an independent relationship with cerebral WMH even after adjustment for atherosclerotic risk factors and the presence of ICAS as confounders, which were reported to be related to WMH in previous reports^10,26,27,41^. Model 1 adjusted for age and sex; model 2 adjusted for age, sex, and atherosclerotic risk factors (BMI, hypertension, diabetes, dyslipidemia, current or former smoker, regular exercise, history of coronary artery disease, and homocysteine level); and model 3 adjusted for age, sex, atherosclerotic risk factors, and presence of ICAS. The presence of cerebral WMH according to the CAC score category was evaluated using a CAC score of 0 as a reference.

Statistical analyses were performed by using Stata version 16.1 (StataCorp, College Station, TX, USA) and R studio version 3.6.3 (RStudio, Boston, MA, USA). A two-tailed *p* value < 0.05 was considered indicative of statistical significance.

## Results

The baseline characteristics of the 1337 individuals are shown in Table 1. The mean age of the participants was evaluated based on brain MRI scan time and was 51.63 ± 9.20 years, and 86.54% of the study population was male. The leading atherosclerotic risk factor in the cohort was current or former smoking history (57.82%), followed by dyslipidemia (51.76%) and hypertension (28.65%). Regarding the radiological variables, 158 subjects (11.82%) had PVWMH, 148 (11.07%) had DWMH, and 21 (1.57%) had ICAS. Regarding the CAC scores, 849 subjects (63.5%) had a CAC score of 0, 332 (24.83%) had a score between 0‒100, and 156 (11.67%) had a score above 100.

**Table 1:** Baseline characteristics of the study participants (n = 1337).

	Missing data, n	
Age, years [SD]	0	51.63 ± 9.20
Male, n (%)	0	1,157 (86.54)
Body mass index, kg/m^2^ [SD]	0	24.88 ± 3.04
**Biochemical variables**		
Systolic blood pressure, mmHg [SD]	0	115.95 ± 12.45
Diastolic blood pressure, mmHg [SD]	0	75.73 ± 9.52
Glucose, mg/dL [SD]	0	101.57 ± 18.01
HbA1c, % [SD]	1	5.73 ± 0.68
Total cholesterol, mg/dL [SD]	0	192.74 ± 38.65
LDL cholesterol, mg/dL [SD]	0	130.98 ± 37.58
HDL cholesterol, mg/dL [SD]	0	54.53 ± 14.13
Triglycerides, mg/dL [SD]	0	138.66 ± 102.11
Homocysteine, μmol/L [SD]	0	10.52 ± 4.51
**Medications**		
Antihypertensive agents, n (%)	0	286 (21.39)
Antidiabetic agents, n (%)	0	103 (7.7)
Lipid-lowering agents, n (%)	0	222 (16.6)
**Risk factors**		
Hypertension, n (%)	0	383 (28.65)
Diabetes, n (%)	0	157 (11.74)
Dyslipidemia, n (%)	0	692 (51.76)
Current or former smoker, n (%)	43	773 (57.82)
Regular exercise^a^, n (%)	25	455 (34.03)
History of CAD, n (%)	0	45 (3.37)
CAC score, median [IQR]	0	0 [0–18]
CAC 0		849 (63.5)
CAC 0‒100		332 (24.83)
CAC > 100		156 (11.67)
**Radiological variables**		
PVWMH, n (%)	0	158 (11.82)
DWMH, n (%)	0	148 (11.07)
ICAS, n (%)	0	21 (1.57)

*PVWMH* Periventricular white matter hyperintensity, *DWMH* Deep white matter hyperintensity, *LDL* Low-density lipoprotein, *HDL* High-density lipoprotein, *HbA1c* Glycohemoglobin, *CAD* Coronary artery disease, *CAC* Coronary artery calcium, *ICAS* Intracranial artery stenosis, *SD* Standard deviation, *IQR* Interquartile range.

^a^Regular engagement in vigorous exercise for more than 10 min at least 3 times per week.

**Figure 2:**
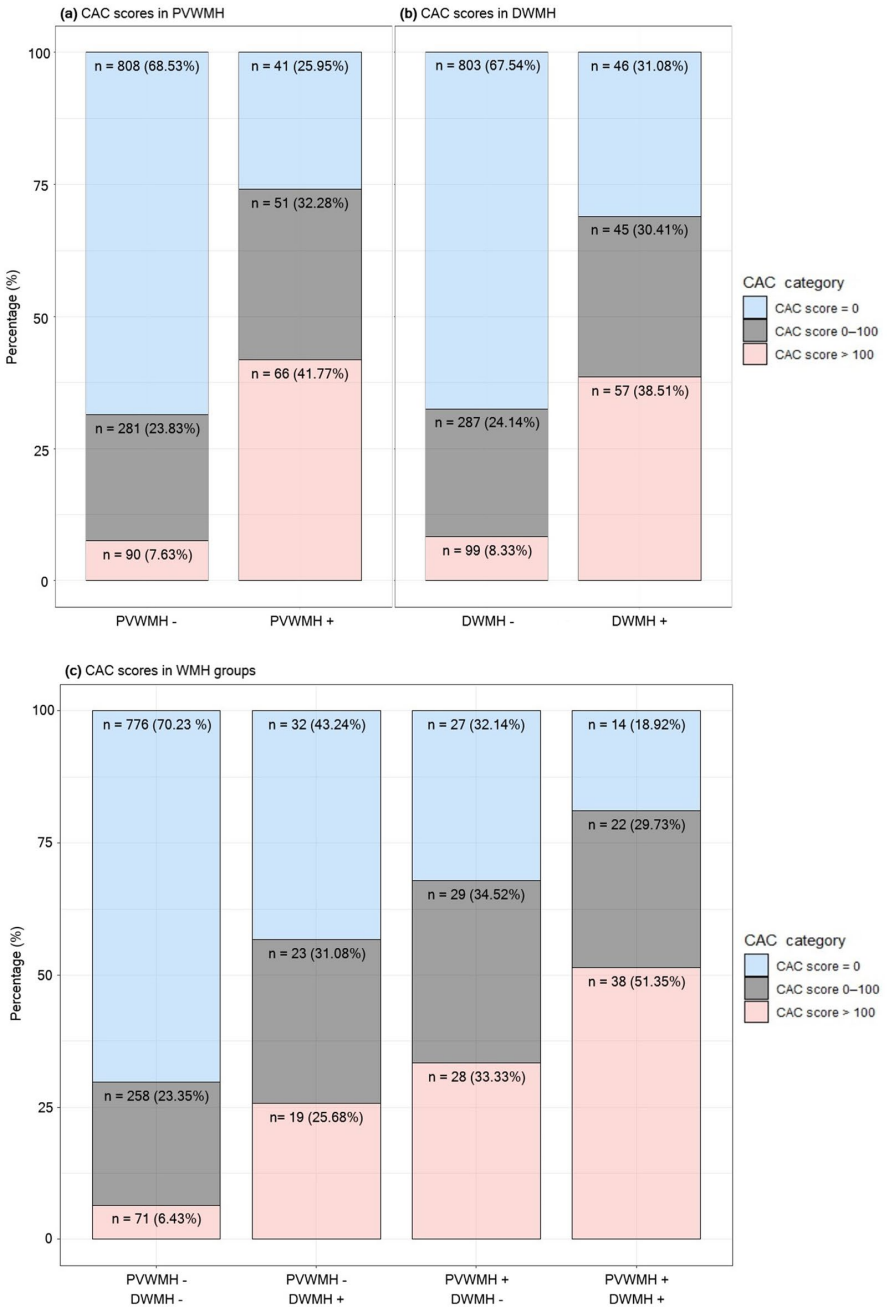
Percentages of CAC score categories according to the presence of PVMWH **(a)**, DWMH **(b)** and PVWMH or DWMH **(c)**. *CAC* Coronary artery calcium, *WMH* White matter hyperintensity, *PVWMH* Periventricular white matter hyperintensity, *DWMH* Deep white matter hyperintensity.

In univariate analysis, age, sex, and the majority of the atherosclerotic risk factors except BMI, dyslipidemia and current or former history of smoking were significantly associated with the presence of cerebral WMH (*p* < 0.05) (Table 2). Individuals with PVWMH and DWMH were older than those without and had a greater burden of hypertension, diabetes, history of coronary artery disease, CAC, and ICAS. In univariate analysis, the percentage of females and subjects who responded that they performed regular exercise was higher in the groups with WMH. The median (interquartile range; IQR) CAC score was 62 (IQR 0‒269.5) in the PVWMH group and 46.5 (IQR 0‒192) in the DWMH group. The distribution of CAC categories according to the presence of PVWMH and DWMH is shown in Fig. 2. The proportion of categories with higher CAC scores increased as the degree of accompanying WMH increased.

**Table 2:** Baseline characteristics of the groups with or without PVWMH and DWMH.

	PVWMH		*p*	DWMH		*p*
	Absent (n = 1179)	Present (n = 158)		Absent (n = 1189)	Present (n = 148)	
**Clinical variables**						
Age, years [SD]	50.22 ± 8.35	62.1 ± 8.47	< 0.001	50.41 ± 8.44	61.39 ± 9.26	< 0.001
Male, n (%)	1037 (87.96)	120 (75.95)	< 0.001	1048 (88.14)	109 (73.65)	< 0.001
BMI, kg/m^2^ [SD]	24.85 ± 3.04	25.09 ± 2.99	0.350	24.84 ± 3.07	25.17 ± 2.77	0.945
Hypertension, n (%)	293 (24.85)	90 (56.96)	< 0.001	302 (25.4)	81 (54.73)	< 0.001
Diabetes, n (%)	114 (9.67)	43 (27.22)	< 0.001	121 (10.18)	36 (24.32)	< 0.001
Dyslipidemia, n (%)	615 (52.16)	77 (48.73)	0.418	615 (51.72)	77 (52.03)	0.945
Current or former smoker, n (%)	691 (58.61)	82 (51.9)	0.080	688 (57.86)	85 (57.43)	0.015
Regular exercise^a^, n (%)	385 (32.65)	70 (44.3)	0.003	398 (33.47)	57 (38.51)	0.003
History of CAD, n (%)	34 (2.88)	11 (6.96)	0.008	33 (2.78)	12 (8.11)	0.002
CAC score, median [IQR]	0 [0‒7]	62 [0‒269.5]	< 0.001	0 [0‒9]	46.5 [0‒192]	< 0.001
**Radiological variables**						
PVWMH, n (%)	–	–		84 (7.06)	74 (50)	< 0.001
DWMH, n (%)	74 (6.28)	74 (46.84)	< 0.001	–	–	
ICAS, n (%)	8 (0.68)	13 (8.23)	< 0.001	7 (0.59)	14 (9.46)	< 0.001

*PVWMH* Periventricular white matter hyperintensity, *DWMH* Deep white matter hyperintensity, *BMI* Body mass index, *LDL* Low-density lipoprotein, *HDL* High-density lipoprotein, *HbA1c* Glycohemoglobin, *CAD* Coronary artery disease, *CAC* Coronary artery calcium, *ICAS* Intracranial artery stenosis, *SD* Standard deviation, *IQR* Interquartile range.

^a^Regular engagement in vigorous exercise for more than 10 min at least 3 times per week.

In multivariable regression analysis, age (OR 1.13; 95% CI 1.10‒1.16, OR 1.11; 95% CI 1.08‒1.14, respectively) and hypertension (OR 2.29; 95% CI 1.50‒3.50, OR 1.98; 95% CI 1.30‒3.02, respectively) were independent significant clinical predictors of PVWMH and DWMH after adjustment for age, sex, atherosclerotic risk factors (BMI, hypertension, diabetes, dyslipidemia, current or former smoker, exercise, history of coronary artery disease, and homocysteine level), and ICAS (all *p* < 0.05) (Table 3). There were no significant associations between WMH and sex, BMI, presence of diabetes or dyslipidemia, history of cigarette smoking or regular exercise after adjustment.

**Table 3:** Multivariable analysis for PVWMH and DWMH after adjusting for confounders.

	Model 1^b^				Model 2^c^				Model 3^d^			
	PVWMH		DWMH		PVWMH		DWMH		PVWMH		DWMH	
	OR (95% CI)	*p*	OR (95% CI)	*p*	OR (95% CI)	*p*	OR (95% CI)	*p*	OR (95% CI)	*p*	OR (95% CI)	*p*
**CAC score**												
0 (reference)	1 (reference)		1 (reference)		1 (reference)		1 (reference)		1 (reference)		1 (reference)	
0–99	2.31 (1.45–3.73)	< 0.001	1.83 (1.15–2.92)	0.011	2.23 (1.37–3.62)	0.001	1.60 (0.99–2.58)	0.057	2.22 (1.36–3.61)	0.001	1.59 (0.98–2.58)	0.061
> 100	6.28 (3.76–10.49)	< 0.001	4.56 (2.74–7.59)	< 0.001	5.76 (3.31–10.05)	< 0.001	3.96 (2.29–6.84)	< 0.001	5.45 (3.11–9.54)	< 0.001	3.66 (2.10–6.38)	< 0.001
Age	1.13 (1.11–1.16)	< 0.001	1.11 (1.09–1.14)	< 0.001	1.13 (1.10–1.16)	< 0.001	1.11 (1.08–1.14)	< 0.001	1.13 (1.10–1.16)	< 0.001	1.11 (1.08–1.14)	< 0.001
Sex	0.86 (0.52–1.42)	0.55	0.69 (0.42–1.13)	0.142	0.88 (0.47–1.63)	0.679	0.60 (0.32–1.13)	0.114	0.84 (0.45–1.56)	0.578	0.55 (0.29–1.04)	0.066
Hypertension					2.34 (1.54–3.57)	< 0.001	2.04 (1.35–3.10)	0.001	2.29 (1.50–3.50)	< 0.001	1.98 (1.30–3.02)	0.001
BMI					1.04 (0.97–1.11)	0.303	1.06 (0.98–1.13)	0.134	1.03 (0.96–1.11)	0.362	1.05 (0.98–1.13)	0.173
Diabetes					1.43 (0.86–2.39)	0.168	1.18 (0.71–1.98)	0.52	1.30 (0.77–2.19)	0.328	1.04 (0.61–1.77)	0.897
Dyslipidemia					0.69 (0.46–1.04)	0.075	0.88 (0.59–1.31)	0.52	0.68 (0.45–1.03)	0.071	0.86 (0.57–1.30)	0.477
Current or former smoker					0.74 (0.46–1.20)	0.225	1.56 (0.93–2.62)	0.09	0.76 (0.47–1.24)	0.269	1.65 (0.97–2.81)	0.062
Regular exercise^a^					0.97 (0.85–1.11)	0.646	1.04 (0.93–1.16)	0.526	0.98 (0.86–1.11)	0.723	1.05 (0.94–1.17)	0.419
History of CAD					0.52 (0.21–1.28)	0.154	0.88 (0.38–2.02)	0.759	0.45 (0.17–1.16)	0.099	0.76 (0.32–1.85)	0.551
Homocysteine					1.01 (0.96–1.07)	0.666	0.92 (0.84–1.10)	0.04	1.02 (0.96–1.07)	0.589	0.92 (0.85–1.00)	0.056
ICAS									3.97 (1.31–12.06)	0.015	7.11 (2.33–21.77)	0.001

*PVWMH* Periventricular white matter hyperintensity, *DWMH* Deep white matter hyperintensity, *ICAS* Intracranial artery stenosis, *CAC* Coronary artery calcium, *CAD* Coronary artery disease, *CI* Confidence interval, *OR* Odds ratio.

^a^Regular engagement in vigorous exercise for more than 10 min at least 3 times per week.

^b^Adjusted for age and sex.

^c^Adjusted for Model 1 + atherosclerotic risk factors (BMI, hypertension, diabetes, dyslipidemia, current or former smoker, regular exercise, history of CAD, and homocysteine level).

^d^Adjusted for Model 2 + ICAS.

The categories of higher CAC scores showed an increased association with cerebral WMH in a dose-dependent relationship compared to the reference category with a CAC score of 0 even after adjusting for the confounding factors. For both PVWMH and DWMH, the category with a CAC score of more than 100 (OR 5.45; 95% CI 3.11‒9.54, OR 3.66; 95% CI 2.10‒6.38, respectively) showed a greater association than the category with a CAC score between 0 and 100 (OR 2.22; 95% CI 1.36‒3.61, OR 1.59; 95% CI 0.98‒2.58, respectively). In comparing the association with CAC between the PVWMH and DWMH groups, all three models of the multivariable analysis revealed a higher correlation with PVWMH in both CAC score categories. The presence of ICAS also showed significant associations with both PVWMH (OR 3.97; 95% CI 1.31‒12.06) and DWMH (OR 7.11; 95% CI 2.33‒21.77).

The variance inflation factor was calculated for all regression models to evaluate potential multicollinearity, and no problematic multicollinearity was found (Supplementary Table 1 online).

## Discussion

In this study, the risk of cerebral WMH increased with higher CAC scores in a dose-dependent relationship, and the results were statistically significant after adjusting for confounding atherosclerotic risk factors. Our findings are in agreement with those of previous studies showing an association between CAC and brain MRI abnormalities, further supporting the association of CAC with small vessels of the brain as well as atherosclerosis of large vessels^29–32^.

Interestingly, the ORs of the CAC scores were slightly higher for the PVWMH group than for the DWMH group in all three models of the multivariable analysis. This difference might be related to the fact that it is presumed that dissimilarity exists among the pathophysiological processes and risk factors between PVWMHs and DWMHs^11,42,43^. PVWMHs are typically symmetrically present in both cerebral hemispheres, which is suggestive of diffuse perfusion disturbances, whereas DWMHs often have an asymmetric distribution, suggesting that they are caused by local perfusion disturbances^44^. Since the periventricular region is supplied by end arteries of long medullary and perforating branches^45^, it is particularly vulnerable to hypoperfusion and ischemia when the autoregulatory mechanism that maintains constant brain perfusion is impaired by arteriosclerosis or lipohyalinosis^46–49^. In particular, several studies have demonstrated that reflections of systemic atherosclerosis, such as hypertension, diabetes and the presence of aortic atherosclerosis, are preferentially associated with PVWMH^50–53^, which supports our study results that the CAC score, age, and hypertension have higher ORs for PVWMH than for DWMH in all models.

In this study, the presence of ICAS was strongly associated with cerebral WMH, and this result can be explained by the fact that significant stenosis of the major intracranial artery reduces local or territorial brain perfusion, and this chronic hypoperfusion promotes lipohyalinosis, which is the mechanism underlying WMH development^26,54^.

In line with many previous studies conducted on different ethnicities^3,27,28,55^, our study also showed that age and hypertension have independent significant associations with cerebral WMH in the multivariable analysis. However, associations between WMH and other atherosclerotic risk factors have shown varying results among previous reports^27,28,37,56^. The reasons for these varying results are likely due to differences in the study population, the criteria for defining the risk factors, or the method used to analyze WMH, which necessitates further studies.

There are several limitations of this study that should be noted. First, this is a retrospective study from an Asian population in single-brand healthcare centers. The risk of selection bias is possible as a significant number of study participants were of working age and more than half were male, which is due to the unique characteristics of Korea that the country requires companies to provide regular health examinations to their employees. To reduce bias in cohort studies, long-term, longitudinal and prospective studies such as the Rotterdam study^57^ or the Framingham study^58^ should be pursued, and there have been many previous reports focusing on the relationship between cerebral WMH and various atherosclerotic risk factors using the Rotterdam and Framingham study cohorts^4,59–63^. However, since none of the existing studies have focused on the association between WMH and CAC in the normal general population, our study results have clinical significance. Second, since the MRI analysis was performed visually by radiologists, the objectivity might be insufficient. However, we sought to overcome this limitation by including a large number of participants and defining subjects with at least moderate or higher WMH as a positive group. In addition, we conducted inter- and intraobserver reliability tests, which showed good agreement. There are also previous reports showing a high correlation between the visual scoring method using the Fazekas scale and volumetry analysis for evaluation of the extent of WMH^64,65^. Third, individuals with brain pathologies were excluded through a self-administered questionnaire regarding past medical history and image analysis in individuals with obvious disease, and it may not have been possible to filter out individuals with subclinical disease. In addition, the brain MRI protocol in our institution for health screening does not include enhanced images, so there is a possibility of missed cases with brain pathology that enhances which is not conspicuous in T1-, T2-weighted and FLAIR images, and accuracy in judging the presence of ICAS would have been relatively low compared to enhanced MRA. Fourth, as the participants of this study were from a healthy general population mostly without any disease, the proportion of subjects with ICAS was relatively small.

Nonetheless, the present study included a larger number of healthy individuals than previous studies aiming to elucidate the association between WMH and CAC, and to our knowledge, this is the first study to include adults from a healthy population without any sex or age restrictions^31,32^.

Since the accessibility of brain imaging and the average life expectancy have significantly increased, the importance of cerebral WMH and various related neurologic diseases such as dementia and stroke is being emphasized, but these diseases remain unconquered. The presence of cerebral WMH lesions is associated with steeper cognitive decline, dementia, depression, and stroke, and there is accumulating evidence that controlling certain atherosclerotic risk factors can prevent the occurrence and progression of WMH^12–23,66–69^. Therefore, the results of our study might provide evidence for referring to the CAC score in screening individuals at risk of cerebral WMH, which is an important risk and prognostic factor for various neurologic diseases, thus identifying patients who can benefit from active diagnostic and therapeutic measures. Future research is warranted to evaluate whether CAC plays a significant and independent role in the development of WMH in longitudinal and prospective studies from various regions, age groups, and ethnicities, and other MRI markers of cerebral small vessel disease should also be incorporated for integrated insight.

In conclusion, the CAC score as well as age and hypertension showed a significant association with cerebral WMH in a large-scale healthy population. The CAC score, an indicator of atherosclerotic burden, has a potential role in predicting individuals with a risk of cerebral WMH in clinical practice.

## Supplementary Information

Supplementary Information.

## Data Availability

The dataset analyzed during this study is not publicly available as it consists of individuals’ sensitive personal information. The data are available from the Total Healthcare Center of Kangbuk Samsung Hospital upon reasonable request from qualified researchers trained in research with human subjects. Every request will be reviewed by the institutional review board of Kangbuk Samsung Hospital, and researchers can access the data according to the approval conditions.
