# Supplementary material for: Association of cerebral white matter hyperintensities with coronary artery calcium in a healthy population: a cross-sectional study
Source: Sci Rep. 2022 Dec 13;12:21562. doi: 10.1038/s41598-022-25654-9 (PMC9747792; doi:10.1038/s41598-022-25654-9)
Supplement: Supplementary file 1 — Supplementary Information. [file 41598_2022_25654_MOESM1_ESM.pdf]

Supplementary data for:

Association of Cerebral White Matter Hyperintensities with Coronary Artery Calcium in a Healthy Population: A Cross-Sectional Study

Jinyoung Choi[1], MD; Jung Youn Kim[2], MD; Heon-Ju Kwon[1], MD, PhD; Hye Jeong Choi[2], MD; Sang Heum Kim[2], MD; Sinae Kim[3]; Jungbin Lee[4], MD; and Ji Eun Park[5], MD, PhD

[1]Department of Radiology, Kangbuk Samsung Hospital, Sungkyunkwan University School of Medicine, Seoul 03181, Republic of Korea

[2]Department of Radiology, CHA Bundang Medical Center, CHA University, Seongnam 13496, Republic of Korea

[3]Division of Biostatistics, Department of R&D Management, Kangbuk Samsung Hospital, Sungkyunkwan University School of Medicine, Seoul 03181, Republic of Korea

[4]Department of Radiology, Soonchunhyang University Bucheon Hospital, Bucheon 14584, Republic of Korea

[5]Department of Radiology and Research Institute of Radiology, University of Ulsan College of Medicine, Asan Medical Center, Seoul 05505, Republic of Korea

Corresponding Author: Jung Youn Kim, M.D.

Department of Radiology, CHA Bundang Medical Center, CHA University, 59 Yatap-ro, Bundang, Seongnam, Gyeonggi-do 13496, Republic of Korea

Phone: 82-31-780-5371

E-mail address: cleopiece@gmail.com

Supplementary Figure:

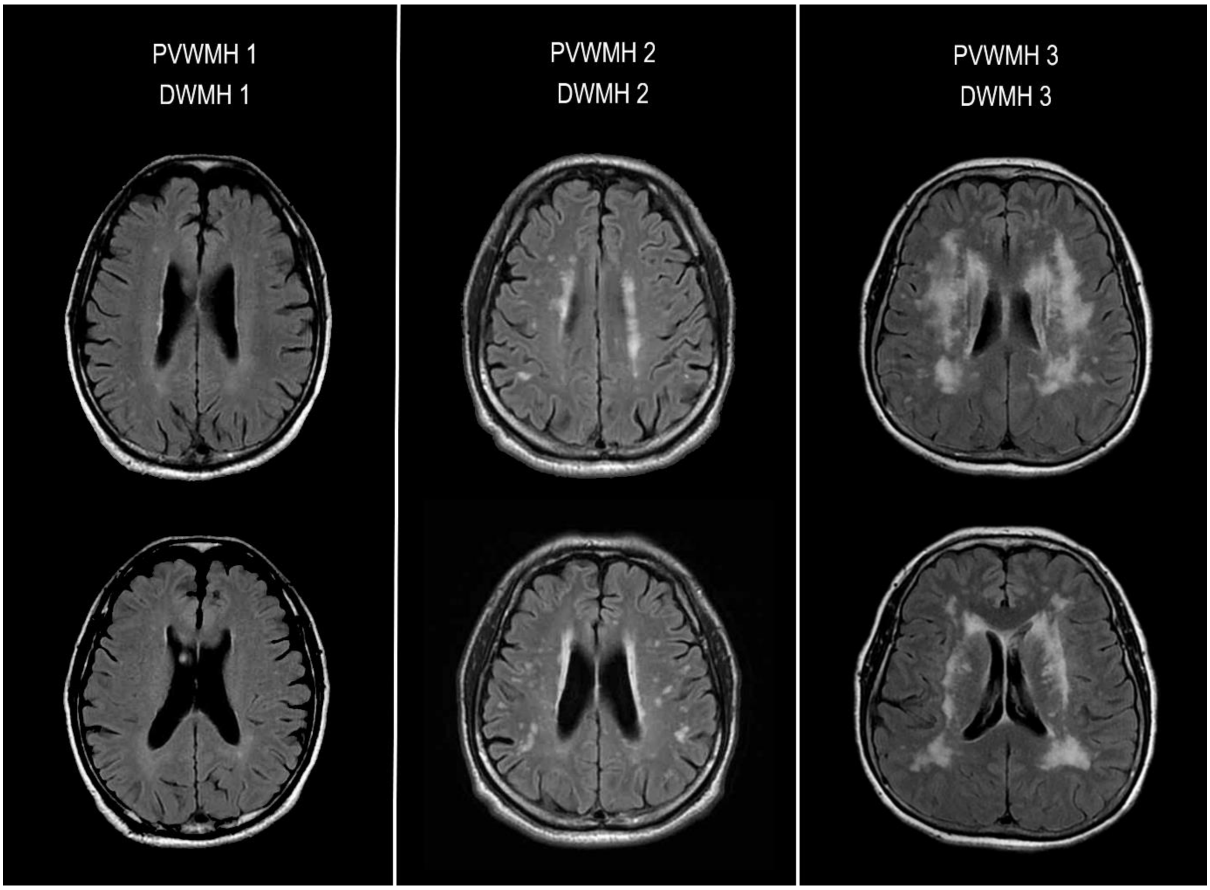

Supplementary Figure 1. Examples of the visual assessment for cerebral white matter hyperintensity according to the Fazekas score. PVWMH indicates periventricular white matter hyperintensity; DWMH, deep white matter hyperintensity.

Supplementary Table:

Supplementary Table 1. The variance inflation factors of the variables in multivariable regression analysis

	Variance inflation factor		
	Model 1[b]	Model 2[c]	Model 3[d]
Age	1.4	1.53	1.54
Sex	1.18	1.49	1.49
CAC score	1.25	1.37	1.39
Body mass index		1.15	1.15
Hypertension		1.16	1.16
Diabetes		1.1	1.12
Dyslipidemia		1.06	1.06
Current or former smoker		1.32	1.33
Regular exercise[a]		1.07	1.07
History of CAD		1.06	1.07
Homocysteine		1.05	1.05
ICAS			1.08

CAC, coronary artery calcium; CAD, coronary artery disease; and ICAS, intracranial artery stenosis.

[a] Regular engagement in vigorous exercise for more than 10 minutes at least 3 times per week.

[b] Adjusted for age and sex.

[c] Adjusted for Model 1 + atherosclerotic risk factors (BMI, hypertension, diabetes, dyslipidemia, current or former smoker, regular exercise, history of CAD, and homocysteine level).

[d] Adjusted for Model 2 + ICAS.
